# Pulsed electric field, cryoballoon, and radiofrequency for paroxysmal atrial fibrillation ablation: a propensity score-matched comparison

**DOI:** 10.1093/europace/euae016

**Published:** 2024-01-20

**Authors:** Domenico G Della Rocca, Lorenzo Marcon, Michele Magnocavallo, Roberto Menè, Luigi Pannone, Sanghamitra Mohanty, Vasileios Sousonis, Antonio Sorgente, Alexandre Almorad, Antonio Bisignani, Andrzej Głowniak, Alvise Del Monte, Gezim Bala, Marco Polselli, Sahar Mouram, Vincenzo Fazia La Fazia, Erwin Ströker, Carola Gianni, Sarah Zeriouh, Stefano Bianchi, Juan Sieira, Stephane Combes, Andrea Sarkozy, Pietro Rossi, Serge Boveda, Andrea Natale, Carlo de Asmundis, Gian-Battista Chierchia, HRMC Investigators, Charles Audiat, Giampaolo Vetta, María Cespón-Fernández, Ioannis Doundoulakis, Cinzia Monaco, Ingrid Overeinder, Gregory Carette, Ilenia Lombardo, Kazutaka Nakasone, Ivan Eltzov, Mark La Meir

**Affiliations:** Heart Rhythm Management Centre, Postgraduate Program in Cardiac Electrophysiology and Pacing, Universitair Ziekenhuis Brussel-Vrije Universiteit Brussel, European Reference Networks Guard-Heart, Laarbeeklaan 101, 1090 Jette, Brussels, Belgium; St. David’s Medical Center, Texas Cardiac Arrhythmia Institute, 3000 N Interstate Hwy 35 Suite 720, Austin, 78705 TX, USA; Heart Rhythm Management Centre, Postgraduate Program in Cardiac Electrophysiology and Pacing, Universitair Ziekenhuis Brussel-Vrije Universiteit Brussel, European Reference Networks Guard-Heart, Laarbeeklaan 101, 1090 Jette, Brussels, Belgium; Arrhythmology Unit, Ospedale Fatebenefratelli Isola Tiberina-Gemelli Isola, Rome, Italy; Heart Rhythm Department, Clinique Pasteur, Toulouse, France; Heart Rhythm Management Centre, Postgraduate Program in Cardiac Electrophysiology and Pacing, Universitair Ziekenhuis Brussel-Vrije Universiteit Brussel, European Reference Networks Guard-Heart, Laarbeeklaan 101, 1090 Jette, Brussels, Belgium; St. David’s Medical Center, Texas Cardiac Arrhythmia Institute, 3000 N Interstate Hwy 35 Suite 720, Austin, 78705 TX, USA; Heart Rhythm Department, Clinique Pasteur, Toulouse, France; Heart Rhythm Management Centre, Postgraduate Program in Cardiac Electrophysiology and Pacing, Universitair Ziekenhuis Brussel-Vrije Universiteit Brussel, European Reference Networks Guard-Heart, Laarbeeklaan 101, 1090 Jette, Brussels, Belgium; Heart Rhythm Management Centre, Postgraduate Program in Cardiac Electrophysiology and Pacing, Universitair Ziekenhuis Brussel-Vrije Universiteit Brussel, European Reference Networks Guard-Heart, Laarbeeklaan 101, 1090 Jette, Brussels, Belgium; Arrhythmology Unit, Ospedale Fatebenefratelli Isola Tiberina-Gemelli Isola, Rome, Italy; Department of Cardiology, Medical University of Lublin, Lublin, Poland; Heart Rhythm Management Centre, Postgraduate Program in Cardiac Electrophysiology and Pacing, Universitair Ziekenhuis Brussel-Vrije Universiteit Brussel, European Reference Networks Guard-Heart, Laarbeeklaan 101, 1090 Jette, Brussels, Belgium; Heart Rhythm Management Centre, Postgraduate Program in Cardiac Electrophysiology and Pacing, Universitair Ziekenhuis Brussel-Vrije Universiteit Brussel, European Reference Networks Guard-Heart, Laarbeeklaan 101, 1090 Jette, Brussels, Belgium; Arrhythmology Unit, Ospedale Fatebenefratelli Isola Tiberina-Gemelli Isola, Rome, Italy; Heart Rhythm Management Centre, Postgraduate Program in Cardiac Electrophysiology and Pacing, Universitair Ziekenhuis Brussel-Vrije Universiteit Brussel, European Reference Networks Guard-Heart, Laarbeeklaan 101, 1090 Jette, Brussels, Belgium; St. David’s Medical Center, Texas Cardiac Arrhythmia Institute, 3000 N Interstate Hwy 35 Suite 720, Austin, 78705 TX, USA; Heart Rhythm Management Centre, Postgraduate Program in Cardiac Electrophysiology and Pacing, Universitair Ziekenhuis Brussel-Vrije Universiteit Brussel, European Reference Networks Guard-Heart, Laarbeeklaan 101, 1090 Jette, Brussels, Belgium; St. David’s Medical Center, Texas Cardiac Arrhythmia Institute, 3000 N Interstate Hwy 35 Suite 720, Austin, 78705 TX, USA; Heart Rhythm Department, Clinique Pasteur, Toulouse, France; Arrhythmology Unit, Ospedale Fatebenefratelli Isola Tiberina-Gemelli Isola, Rome, Italy; Heart Rhythm Management Centre, Postgraduate Program in Cardiac Electrophysiology and Pacing, Universitair Ziekenhuis Brussel-Vrije Universiteit Brussel, European Reference Networks Guard-Heart, Laarbeeklaan 101, 1090 Jette, Brussels, Belgium; Heart Rhythm Department, Clinique Pasteur, Toulouse, France; Heart Rhythm Management Centre, Postgraduate Program in Cardiac Electrophysiology and Pacing, Universitair Ziekenhuis Brussel-Vrije Universiteit Brussel, European Reference Networks Guard-Heart, Laarbeeklaan 101, 1090 Jette, Brussels, Belgium; Arrhythmology Unit, Ospedale Fatebenefratelli Isola Tiberina-Gemelli Isola, Rome, Italy; Heart Rhythm Department, Clinique Pasteur, Toulouse, France; St. David’s Medical Center, Texas Cardiac Arrhythmia Institute, 3000 N Interstate Hwy 35 Suite 720, Austin, 78705 TX, USA; Case Western Reserve University School of Medicine, Health Education Campus, 9501 Euclid Ave, Cleveland, 44106 OH, USA; Heart Rhythm Management Centre, Postgraduate Program in Cardiac Electrophysiology and Pacing, Universitair Ziekenhuis Brussel-Vrije Universiteit Brussel, European Reference Networks Guard-Heart, Laarbeeklaan 101, 1090 Jette, Brussels, Belgium; Heart Rhythm Management Centre, Postgraduate Program in Cardiac Electrophysiology and Pacing, Universitair Ziekenhuis Brussel-Vrije Universiteit Brussel, European Reference Networks Guard-Heart, Laarbeeklaan 101, 1090 Jette, Brussels, Belgium

**Keywords:** Atrial fibrillation, Pulsed field ablation, Cryoablation, Radiofrequency, Atrial flutter, Focal ablation, Single-shot

## Abstract

**Aims:**

Pulsed field ablation (PFA) has emerged as a novel, non-thermal energy source to selectively ablate cardiac tissue. We describe a multicentre experience on pulmonary vein isolation (PVI) via the pentaspline Farapulse™ PFA system vs. thermal-based technologies in a propensity score-matched population of paroxysmal atrial fibrillation (PAF) patients.

**Methods and results:**

Propensity score matching was adopted to compare PVI-only ablation outcomes via the Farawave™ system (Group PFA), cryoballoon (Group CRYO), or focal radiofrequency (Group RF) (PFA:CRYO:RF ratio = 1:2:2). Among 1572 (mean age: 62.4 ± 11.3 years; 42.5% females) PAF patients undergoing first time PVI with either PFA (*n* = 174), CRYO (*n* = 655), or RF (*n* = 743), propensity score matching yielded 174 PFA, 348 CRYO, and 348 RF patients. First-pass isolation was achieved in 98.8% of pulmonary veins (PVs) with PFA, 81.5% with CRYO, and 73.1% with RF (*P* < 0.001). Procedural and dwell times were significantly shorter with PFA, whereas the availability of a 3D mapping system led to a significant reduction in X-ray exposure with RF. Overall complication rates were 3.4% (*n* = 6) with PFA, 8.6% (*n* = 30) with CRYO, and 5.5% (*n* = 19) with RF (*P* = 0.052). The 1-year Kaplan–Meier estimated freedom from any atrial tachyarrhythmia was 79.3% with PFA, 74.7% with CRYO, and 72.4% with RF (log-rank *P*-value: 0.24). Among 145 repeat ablation procedures, PV reconnection rate was 19.1% after PFA, 27.5% after CRYO, and 34.8% after RF (*P* = 0.01).

**Conclusion:**

Pulsed field ablation contributed to significantly shorter procedural times. Follow-up data showed a similar arrhythmia freedom, although a higher rate of PV reconnection was documented in post-CRYO and post-RF redo procedures.

## Graphical Abstract

**Graphical Abstract:**
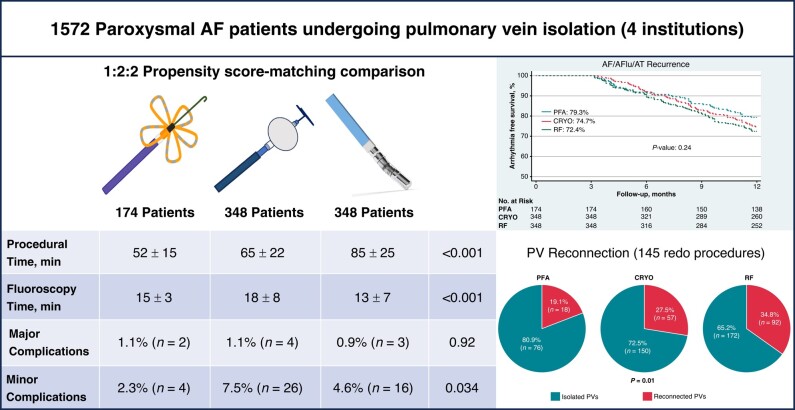

**What’s new?**

- Single-shot pulsed field ablation (PFA) yielded significantly shorter procedural and LA dwelling times, compared to CRYO and radiofrequency (RF). However, 3D-electroanatomical mapping to guide RF-based procedures contributed to significantly shorter exposure to fluoroscopy.
- Pulmonary vein isolation was successfully achieved in all 870 patients; however, first-pass isolation rate was higher with PFA.
- All ablation strategies showed a low risk of serious complications, ranging between 0.9% and 1.1%. Pulsed field ablation patients trended towards lower overall complication rates (*P* = 0.052); this difference was mainly driven by a significantly higher rate of minor complications among CRYO and RF patients (*P* = 0.032).
- Long-term arrhythmia freedom was high (>70% at 1 year) and not significantly different among technologies. Pulsed field ablation patients exhibited a trend towards a lower rate of AF as recurrent arrhythmia.
- At the time of repeat ablation, PFA patients showed a significantly lower number of reconnected PVs compared to those initially treated with CRYO or RF (*P* = 0.01).

## Introduction

Electrical isolation of the pulmonary veins (PVs) is the mainstay strategy of any atrial fibrillation (AF) ablation procedure, either percutaneous or surgical. Percutaneous PV isolation (PVI) has been traditionally performed via focal or single-shot ablation catheters, which rely on thermal energy sources [e.g. radiofrequency (RF) and cryothermy] to promote cardiac tissue ablation.^1,2^ However, achieving safe and effective PVI via thermal-based technologies might be challenging, as a result of their lack of tissue selectivity and a high rate of PV reconnection. Specifically, RF and cryoballoon ablation (CRYO) devices carry a low, but non negligible, risk of collateral damage to adjacent tissues [e.g. phrenic nerve (PN) and oesophagus] and the rate of PV reconnection has been reported to be up to 70%,^3–7^ although this rate is likely lower with new generation ablation systems.

In recent years, a novel energy delivery modality, namely pulsed field ablation (PFA), has emerged as a potential alternative to RF and CRYO for PVI procedures.^8–10^ Pulsed field ablation utilizes ultrashort, high-amplitude electrical pulses to create non-thermal tissue ablation. The underlying mechanism ultimately resulting in cardiac cell death is known as irreversible electroporation, such as a prolonged membrane permeabilization that leads to cell homeostasis disruption beyond repair. As such, cell death occurs due to cardiomyocyte cell physiology impairment rather than a thermal process (tissue heating or freezing).

Farapulse™ (Boston Scientific Inc., Marlborough, MA, USA) is the first PFA technology to have received regulatory approval (CE Mark) and includes the pentaspline PFA catheter Farawave™, which delivers a proprietary biphasic, bipolar waveform of up to 2.0 kV voltage. This system is currently the most researched PFA device, its safety and efficacy being demonstrated in several phase 3 trials and multicentre prospective registries.^9,11–15^ Recently, the randomized ADVENT trial^10^ showed non-inferiority of PFA compared to RF and CRYO with regard to serious adverse events, as well as a composite endpoint of acute procedural success and arrhythmia freedom. Nonetheless, our knowledge on the performance of PFA compared to pre-existing, thermal-based ablation strategies is still limited.^10,12^

Herein we report a real-world, multicentre experience on the procedural success and long-term outcomes of the Farapulse™ PFA system vs. CRYO and RF catheter ablation in a propensity score-matched population of paroxysmal AF (PAF) patients.

## Methods

### Study design

We enrolled consecutive patients undergoing first time catheter ablation of non-valvular PAF. Paroxysmal atrial fibrillation was defined, according to the 2020 ESC Guidelines for the diagnosis and management of AF,^16^ as AF that terminates spontaneously or with intervention within 7 days of onset. Patients with non-PAF, severe valvular heart disease, and hypertrophic cardiomyopathy were excluded. Procedures were performed between June 2021 and September 2022 by 19 different operators at four different centres (Heart Rhythm Management Center, Brussel; St. David’s Medical Center, Austin; Heart Rhythm Department, Clinique Pasteur, Toulouse; Arrhythmology Unit, Ospedale Fatebenefratelli Isola Tiberina-Gemelli Isola, Rome).

For the purpose of the study, PVI-only ablation outcomes by means of the Farawave™ PFA system (Group PFA), a CRYO device (Group CRYO), or focal RF energy using electroanatomical mapping (Group RF) were considered.

A propensity score matching technique was adopted to attenuate the imbalance of covariates among the three groups (PFA:CRYO:RF ratio = 1:2:2).

This multicentre analysis was designed as a retrospective review of individual prospective registries maintained at each participating centre. Individual registries were approved by the local institutional review board. All participants gave written informed consent for each interventional procedure and data collection. The data underlying this article will be shared on reasonable request to the corresponding author.

### Procedural details

All procedures were conducted under general anaesthesia and uninterrupted oral anticoagulation. Anti-arrhythmic drug (AAD) therapy, except amiodarone, was discontinued at least five half-lives before ablation. Heparin was administered before transseptal access and during the procedure, when needed, to maintain the activated clotting time between 300 and 350 s.

#### Pulsed field ablation (Group PFA)

Two ultrasound-guided right groin accesses were used to achieve transseptal access via an 8.5 F SL0 fixed sheath (Abbott, St Paul, MN, USA) and advance a 10-pole catheter into the coronary sinus (CS) or the right ventricle to be used for pacing in case of PFA-related bradycardia. The PFA system has been previously described elsewhere,^9^ and consists of the Farastar™ pulse generator, a dedicated 13 F steerable sheath (Faradrive™), and the PFA catheter Farawave™, an over-the-wire device characterized by five splines and 20 electrodes, which can be deployed into two different configurations (basket and flower). Once transseptal access was achieved, the 8.5 F SL0 fixed sheath was exchanged with the Faradrive™ sheath and the Farapulse™ system (Boston Scientific Inc., Marlborough, MA, USA) was used to achieve PVI.

At least four pairs of PFA applications (two pairs in basket and two pairs in flower configuration, each pair at ∼36° rotation from the other) were performed to achieve PVI. In case of a common PV ostium, a segmental superior and inferior full PFA application set was delivered.

#### CRYO ablation (Group CRYO)

Our cryoballoon ablation protocol has been extensively described in previous publications.^17–19^ Two ultrasound-guided right groin accesses were used to achieve transseptal access via an 8.5 F SL0 fixed sheath (Abbott, St Paul, MN, USA) and advance a 10-pole catheter into the CS or the superior vena cava during right PV ablation in order to monitor diaphragmatic capture via pacing of the ipsilateral phrenic nerve (1000 ms cycle, 20 mA output). The following CRYO technologies were used: Arctic Front Advance PRO, Medtronic Inc., Minneapolis, MN, USA and POLARx Balloon Catheter, Boston Scientific, Marlborough, MA, USA. After transseptal access was achieved, the SL0 sheath was exchanged with a dedicated steerable sheath that was used to advance the CRYO device into the left atrium. Pulmonary vein occlusion was documented via contrast injection through the distal CRYO lumen. Freeze duration was set at 240 s; alternatively, a 180 s freeze was adopted if time to isolation was achieved within 60 s with a temperature <−40°C. In case of a common PV ostium, a segmental superior and inferior full CRYO application set was delivered.

#### Focal radiofrequency ablation (Group RF)

Our focal RF ablation protocol has been described in previous publications.^20–22^ Three right groin accesses under ultrasound guidance were used to achieve double transseptal access via an 8.5 F SL0 fixed sheath (Abbott, St Paul, MN, USA) and advance a 10-pole catheter into the CS. A 3D-electroanatomical mapping system (Carto 3, Biosense Webster, Diamond Bar, CA, USA) was used to guide mapping and ablation. Left atrial electroanatomical mapping and wide antral circumferential ablation (WACA) were achieved via a multipolar catheter (Pentaray) and an open-irrigated RF ablation catheter (Thermocool SmartTouch Surround Flow, Biosense Webster, Diamond Bar, CA, USA). Radiofrequency ablation was performed with a power of 45 W, a desired contact force of 10–15 g, and a target ablation index of 500 for anterior and 400–450 for posterior PV segments. Oesophageal temperature was monitored throughout the procedure via a multi-sensor Circa S-Cath™ oesophageal temperature probe (Circa, Scientific Inc., CO, USA).

### Post-procedural management and follow-up

Protamine was administered at the end of the procedure and haemostasis achieved with manual compression, figure-of-eight suture, or vascular closure device placement,^23^ followed by 3–6 h of bed rest. Patients were discharged after overnight observation, if no periprocedural complications were observed. Anti-arrhythmic drugs were resumed before hospital discharge, maintained for the first 3 months post-ablation (blanking period), and subsequently discontinued if no arrhythmic recurrences were documented during the blanking period. Follow-up visits were scheduled in the outpatient clinic at 1, 3, 6 months, and every 6 months thereafter. Each visit included physical examination, 12-lead ECG, and 24 h Holter monitoring. Alternatively, a 7-day Holter monitoring was prescribed in the presence of symptoms suggestive of arrhythmia recurrence. Patients were also contacted by our staff on a regular basis, as well as instructed to contact the arrhythmia service if new-onset arrhythmic symptoms were noted. In this instance, an in-person examination, inclusive of a 7-day Holter monitoring, was scheduled.

### Definitions and study endpoints

Procedural and dwelling times were measured from femoral puncture to catheter removal and as the time the ablation catheter remained in the left atrium, respectively.

First-pass isolation was defined as successful PV isolation after four pairs of PFA applications in basket and flower configuration (Group PFA), a single cryoballoon application (Group CRYO), or at the completion of WACA (Group RF).

Primary efficacy endpoint was defined as freedom from any atrial tachyarrhythmia >30 s off AADs and occurring after the 3-month blanking period. Primary safety endpoint included any major technology- and procedure-related complications occurring within 7 days post-ablation (except for atrioesophageal fistula).^24^

### Statistical analysis

Normal distribution of all continuous variables was checked by visual methods (Q–Q plot and histogram) and by significance test (Kolmogorov–Smirnov normality test and Shapiro–Wilk’s test). Categorical and continuous data were reported as absolute values (percentage) and mean ± standard deviation or median and interquartile range for non-normal data. Comparisons among groups were performed using Pearson’s bivariate test and χ^2^ tests for categorical covariates; non-parametric test of Kruskal–Wallis was used to compare non-normally distributed continuous variable. All tests were two-sided, and a *P*-value of <0.05 was considered statistically significant. Propensity score matching was performed with a 1:2:2 ratio to reduce the imbalance of covariates among groups. The model considered the following covariates: age, gender, hypertension, diabetes, heart failure, coronary/peripheral artery disease, history of thromboembolic event, chronic kidney disease, and left atrial size. Matching was performed using the nearest neighbour matching protocol (matching ratio of 1 to 2 without replacement) and a calliper width of 0.01. The balance of characteristics was assessed by estimating standardized differences between groups. Analyses were performed with R (Version 4.3.1) and MatchIt package and STATA 18.0 (StataCorp, College Station, TX, USA).

## Results

The study included 1572 (mean age: 62.4 ± 11.3 years; 42.5% females) PAF patients who received first time PVI with either PFA (*n* = 174), CRYO (*n* = 655), or RF (*n* = 743) at four different institutions during a 16-month study period (June 2021–September 2022). Baseline characteristics of the unmatched population are reported in *Table 1*. Propensity score matching based on nine demographic and clinical variables yielded 174 PFA patients, 348 CRYO patients, and 348 RF patients (ratio 1:2:2). Demographics of the matched population are reported in *Table 2*. A well-balanced covariate distribution among groups was observed after propensity matching.

**Table 1:** Baseline characteristics of the unmatched population

Characteristic	PFA *n* = 174	CRYO *n* = 655	RF *n* = 743	*P*-value
Age, years	62 ± 11.6	61.3 ± 13.0	64.3 ± 11.6	0.68
Female gender, *n* (%)	64 (36.8)	264 (40.3)	356 (47.9)	**0**.**003**
BMI, kg/m^2^	27 ± 4.8	27.4 ± 5.0	28.3 ± 5.1	0.46
Hypertension, *n* (%)	78 (44.8)	258 (39.4)	391 (52.6)	**0**.**001**
Diabetes, *n* (%)	16 (9.2)	55 (8.4)	91 (12.2)	**0**.**05**
Heart failure, *n* (%)	13 (7.5)	69 (10.5)	71 (9.6)	0.47
Coronary artery disease, *n* (%)	10 (5.7)	64 (9.8)	112 (15.1)	**0**.**001**
Left atrial diameter, mm	41.8 ± 4.9	41.3 ± 4.8	41.9 ± 4.2	0.43
LVEF, %	59.4 ± 4.2	56.4 ± 9.1	57.4 ± 8.3	0.32
Stroke/TIA, *n* (%)	8 (4.6)	23 (3.5)	41 (5.5)	0.20
CKD, *n* (%)	9 (5.2)	44 (6.7)	72 (9.7)	**0**.**04**
CHA_2_DS_2_-VASC score	2 [1–3]	2 [1–3]	2 [1–3]	0.61

Continuous variables are shown as mean ± standard deviation (SD) or median and interquartile range (IQR). Discrete variables are presented as numbers and percentages (%). Significant values are reported in bold.

AAD, anti-arrhythmic drugs; BMI, body mass index; CKD, chronic kidney disease; CRYO, cryoablation; LVEF, left ventricular ejection fraction; PFA, pulsed field ablation; RF, radiofrequency.

**Table 2:** Baseline characteristics of the propensity score-matched population

Characteristic	PFA *n* = 174	CRYO *n* = 348	RF *n* = 348	*P*-value
Age, years	62 ± 11.6	62.1 ± 12.3	62.9 ± 10.1	0.49
Female gender, *n* (%)	64 (36.8)	130 (37.4)	131 (37.6)	0.98
BMI, kg/m^2^	27 ± 4.8	27.2 ± 4.6	25.8 ± 6.7	0.42
Hypertension, *n* (%)	78 (44.8)	150 (43.1)	146 (42)	0.82
Diabetes, *n* (%)	16 (9.2)	24 (6.9)	34 (9.8)	0.37
Heart failure, *n* (%)	13 (7.5)	31 (8.9)	29 (8.3)	0.86
Coronary artery disease, *n* (%)	10 (5.7)	24 (6.9)	22 (6.3)	0.87
Left atrial diameter, mm	41.8 ± 4.9	41.9 ± 4.9	41.7 ± 4.4	0.55
LVEF, %	59.4 ± 4.2	58.2 ± 7.5	57.9 ± 6.9	0.59
Stroke/TIA, *n* (%)	8 (4.6)	17 (4.9)	14 (4.0)	0.86
CKD, *n* (%)	9 (5.2)	23 (6.6)	18 (5.2)	0.67
CHA_2_DS_2_-VASC score	2 [1–3]	2 [1–3]	2 [1–3]	0.73

Continuous variables are shown as mean ± standard deviation (SD) or median and interquartile range (IQR). Discrete variables are presented as numbers and percentages (%).

AAD, anti-arrhythmic drugs; BMI, body mass index; CKD, chronic kidney disease; CRYO, cryoablation; LVEF, left ventricular ejection fraction; PFA, pulsed field ablation; RF, radiofrequency.

### Procedural details

Complete PV isolation was successful in all 870 propensity score-matched patients; among them, 59 (6.8%) and 6 (0.7%) patients had a left common or right common PV, respectively (*P* = 0.84 for common ostia distribution among groups).

First-pass PV isolation was achieved in 675/683 (98.8%) PVs in Group PFA, 1115/1368 (81.5%) PVs in Group CRYO, and 509/696 (73.1%) ipsilateral PVs in Group RF (*P* < 0.001).

In Group PFA, additional applications to optimize antral isolation were delivered in 37 (21.2%) patients, leading to a mean final number of 34.3 ± 2.1 applications to the PVs. A 35 mm PFA catheter was the device of choice in 6 (3.5%) cases.

In CRYO group, the average number of applications per PV was 1.3 ± 0.6.

Procedural details are depicted in *Table 3*. Procedural and dwell times were significantly shorter with PFA, whereas RF patients showed a significantly shorter fluoroscopy time (*Table 3*). Figure-of-eight suture or vascular closure devices were used in 12.1%, 13.1%, and 15.7% of PFA, CRYO, and RF patients, respectively (*P* = 0.45).

**Table 3:** Procedural details and complications of the propensity score-matched population

Characteristic	PFA *n* = 174	CRYO *n* = 348	RF *n* = 348	*P*-value
Procedural time, min	52.1 ± 14.6	64.5 ± 21.8	84.8 ± 24.8	**<0**.**001**
LA dwell time, min	36.4 ± 12.8	46.2 ± 14.1	64.8 ± 20.9	**<0**.**001**
Fluoroscopy time, min	14.8 ± 3.4	17.6 ± 8.1	12.9 ± 6.9	**<0**.**001**
Total complications, *n* (%)	6 (3.4)	30 (8.6)	19 (5.5)	0.052
Major complications	2 (1.1)	4 (1.1)	3 (0.9)	0.92
Death, *n* (%)	—	—	—	—
AE fistula, *n* (%)	—	—	—	—
PV stenosis, *n* (%)	—	—	—	—
Stroke/TIA/CVA, *n* (%)	*1 (0.6)*	—	*1 (0.3)*	*0*.*61*
Cardiac tamponade, *n* (%)	—	*1 (0.3)*	*1 (0.3)*	*1*
Persistent PN injury, *n* (%)	—	*1 (0.3)*	—	—
Coronary artery spasm, *n* (%)	—	—	—	—
Major vascular complications, *n* (%)^a^	*1 (0.6)*	*2 (0.6)*	*1 (0.3)*	*0*.*82*
Minor complications	4 (2.3)	26 (7.5)	16 (4.6)	**0**.**034**
Transient PN injury, *n* (%)	—	*17 (4.8)*	—	—
Pericarditis, *n* (%)	—	*3 (0.9)*	*11 (3.2)*	***0***.***03***
Groin haematoma, *n* (%)	*4 (2.3)*	*6 (1.7)*	*5 (1.4)*	*0*.*77*

Continuous variables are shown as mean ± standard deviation (SD). Discrete variables are presented as numbers and percentages (%). Significant values are reported in bold.

AE, atrioesophageal; CRYO, cryoballoon ablation; CVA, cerebrovascular accident; PFA, pulsed field ablation; PN, phrenic nerve; PV, pulmonary vein; RF, radiofrequency; TIA, transient ischaemic attack.

^a^Requiring percutaneous or surgical intervention.

Overall complication rates were 3.4% (*n* = 6) with PFA, 8.6% (*n* = 30) with CRYO, and 5.5% (*n* = 19) with RF (*P* = 0.052). This difference was statistically significant when comparing Group PFA vs. Group CRYO (PFA vs. CRYO: *P* = 0.03; PFA vs. RF: *P* = 0.39; CRYO vs. RF: *P* = 0.14). A statistically significant difference was also observed among groups for minor complications (PFA: 2.3% vs. CRYO: 7.5% vs. RF: 4.6%; *P* = 0.034), with transient PN injury (4.8%) being the main determinant of this difference.

Major periprocedural complication rates were similar (*P* = 0.92; *Table 3*). A detailed list of major and minor complications is depicted in *Table 3*.

### Efficacy endpoint

Arrhythmia recurrence during the blanking period occurred in 29 (16.7%) PFA, 63 (18.1%) CRYO, and 78 (22.4%) RF patients (*P* = 0.21; *Table 4*). No significant differences were documented in the type of recurrent arrhythmia (AF vs. atrial flutter/atrial tachycardia) during the blanking period.

**Table 4:** Follow-up characteristics of the propensity score-matched population

Characteristic	PFA *n* = 174	CRYO *n* = 348	RF *n* = 348	*P*-value
Blanking period				
Arrhythmia recurrence, *n* (%)	29 (16.7)	63 (18.1)	78 (22.4)	0.21
AF, *n* (%)	*20 (11.5)*	*49 (14.1)*	*63 (18.1)*	*0.11*
AFlu/AT, *n* (%)	*9 (5.2)*	*14 (4.0)*	*15 (4.3)*	*0.83*
Follow-up				
Arrhythmia recurrence, *n* (%)	36 (20.7)	88 (25.3)	96 (27.6)	0.23
AF, *n* (%)	*25 (14.4)*	*75 (21.6)*	*78 (22.4)*	*0.08*
AFlu/AT, *n* (%)	*11 (6.3)*	*13 (3.7)*	*18 (5.2)*	*0.40*
Redo procedures, *n* (%)	24 (66.7)	53 (60.0)	68 (70.8)	—
Patients with durable PVI, *n* (%)	13 (54.2)	24 (45.3)	23 (33.8)	0.17
Patients with 1 Rec. PV, *n* (%)	4 (16.7)	9 (17.0)	16 (23.5)	—
Patients with 2 Rec. PVs, *n* (%)	7 (29.1)	14 (26.4)	16 (23.5)	—
Patients with 3 Rec. PVs, *n* (%)	—	4 (7.5)	8 (11.8)	—
Patients with 4 Rec. PVs, *n* (%)	—	2 (3.8)	5 (7.4)	—
Follow-up duration, m	12.3 ± 2.1	12.1 ± 2.4	12.6 ± 2.3	0.43

Discrete variables are presented as numbers and percentages (%). Continuous variables are shown as mean ± standard deviation (SD).

AF, atrial fibrillation; AFlu, atrial flutter; AT, atrial tachycardia; CRYO, cryoballoon ablation; PFA, pulsed field ablation; PV, pulmonary vein; Rec., reconnected; RF, radiofrequency.

**Figure 1:**
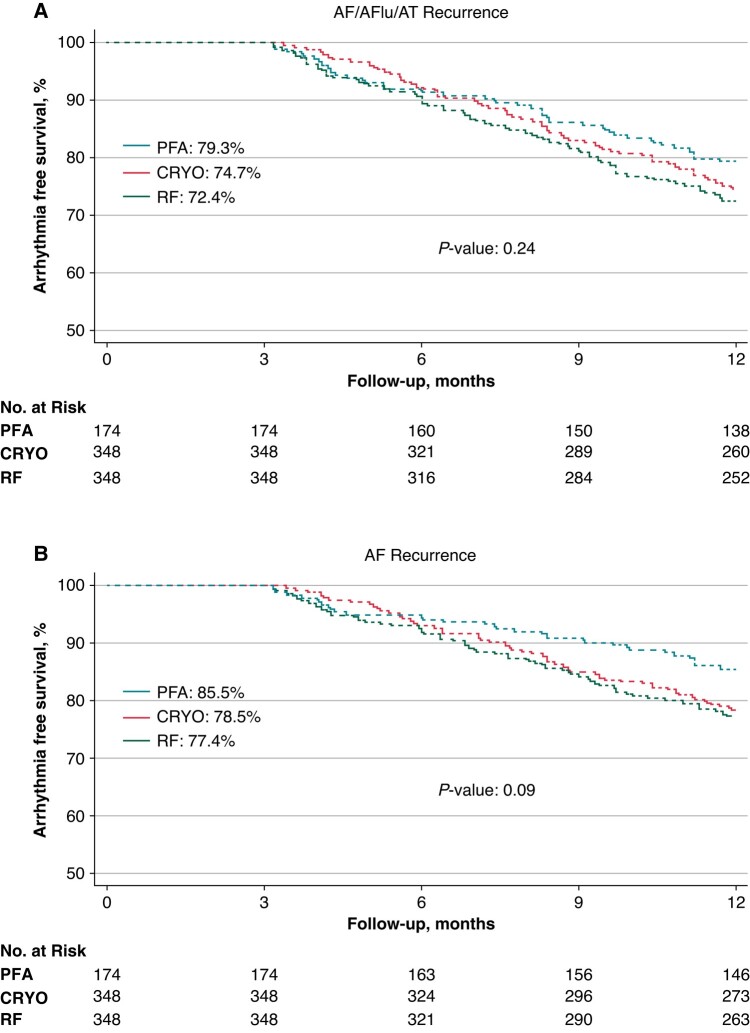
Kaplan–Meier analysis. The graph shows Kaplan–Meier estimates of freedom from any atrial tachyarrhythmias (AF, AFlu, AT; panel *A*) and from documented AF (panel *B*) episodes lasting >30 s after a single procedure and off AAD therapy. AAD, anti-arrhythmic drug; AF, atrial fibrillation; AFlu, atrial flutter; AT, atrial tachycardia; CRYO, cryoballoon; PFA, pulsed field ablation; RF, radiofrequency.

Mean follow-up duration was 12.3 ± 2.3 months (*P* = 0.43). The 1-year Kaplan–Meier estimated freedom from any atrial tachyarrhythmia was 79.3% with PFA, 74.7% with CRYO, and 72.4% with RF (log-rank *P*-value: 0.24; *Figure 1A*). A trend towards a higher freedom from AF was observed after PFA (PFA: 85.5% vs. CRYO: 78.5% vs. RF: 77.4%; log-rank *P* = 0.09; *Figure 1B*).

### Redo procedures and pulmonary vein reconnection

Overall, 145 (65.9%) of 220 patients with arrhythmia relapse underwent repeat ablation (*Tables 4* and *5*).

**Table 5:** Reconnected PVs at the time of redo ablation

Characteristic	PFA *n* = 24	CRYO *n* = 53	RF *n* = 68	*P*-value
PV reconnection, *n*/PVs (%)	18/94 (19.1)	57/207 (27.5)	92/264 (34.8)	**0**.**01**
Median [IQR]	0 [0–2]	1 [0–2]	1 [0–2]	0.14
LSPV, *n* (%)	6/22 (27.3)	8/48 (16.7)	22/62 (35.5)	0.09
LIPV, *n* (%)	3/22 (13.6)	13/48 (27.0)	18/62 (29.0)	0.35
RSPV, *n* (%)	4/24 (16.7)	9/53 (16.9)	21/66 (31.8)	0.11
RIPV, *n* (%)	4/24 (16.7)	24/53 (45.3)	28/66 (42.4)	**0**.**04**
LCPV, *n* (%)	1/2 (50.0)	3/5 (60.0)	2/6 (33.3)	0.8
RCPV, *n* (%)	0/0 (0.0)	0/0 (0.0)	1/2 (50.0)	—

Discrete variables are presented as numbers and percentages (%). Continuous variables are shown as mean ± standard deviation (SD) or median and interquartile range (IQR). Significant values are reported in bold.

CRYO, cryoballoon ablation; LCPV, left common pulmonary vein; LIPV, left inferior pulmonary vein; LSPV, left superior pulmonary vein; PFA, pulsed field ablation; PV, pulmonary vein; RCPV, right common pulmonary vein; RF, radiofrequency; RIPV, right inferior pulmonary vein; RSPV, right superior pulmonary vein.

**Figure 2:**
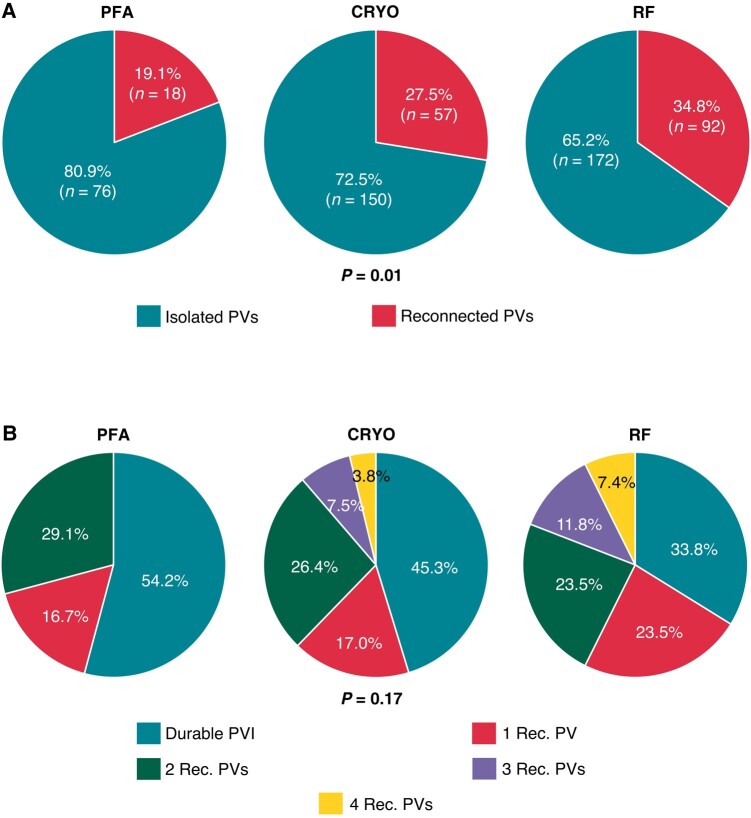
Details on PV reconnection identified during redo procedures. (*A*) Percentage of PVs durably isolated or reconnected according to patient group. (*B*) Percentage of patients with durable PVI vs. those with one to four reconnected PVs per patient according to patient group. CRYO, cryoballoon; PFA, pulsed field ablation; PV, pulmonary vein; PVI, pulmonary vein isolation; Rec., reconnected; RF, radiofrequency.

Pulmonary vein reconnection rate was 18/94 (19.1%) in PFA Group, 57/207 (27.5%) in CRYO Group, and 92/264 (34.8%) in RF Group; the difference was statistically significant (*P* = 0.01; *Tables 4* and *5*; *Figure 2*).

Among the 24 PFA patients undergoing redo ablation, 13 (54.2%) patients had no PV reconnection, whereas 4 (16.7%) and 7 (29.1%) others had one or two reconnected veins, respectively.

Further details on presence and distribution of PV reconnection after PFA, CRYO, and RF are reported in *Tables 4* and *5* and *Figure 2A* and *B*.

The most common site of reconnection was the left superior PV (27.3%) for PFA and the right inferior PV for both CRYO and RF (45.3% and 42.4%, respectively). A trend towards fewer reconnected left superior PVs was observed after CRYO, compared to PFA and RF (16.7% vs. 27.3% vs. 35.5%; *P* = 0.09). No differences in left inferior and right superior PV reconnection rates were documented among groups. Pulsed field ablation patients had a significantly lower rate of right inferior PV reconnection, compared to CRYO and RF (16.7% vs. 45.3% vs. 42.4%; *P* = 0.04).

## Discussion

Herein we describe a real-world multicentre experience on procedural characteristics, arrhythmia-free survival, and findings at repeat ablation in a large multicentre, prospective cohort of PAF undergoing a PVI-only percutaneous procedure via PFA, cryoballoon, or focal RF ablation. Propensity score matching was adopted to attenuate any imbalance of covariates among the three groups.

Our main findings are the following:

1. Single-shot PFA yielded significantly shorter procedural and LA dwelling times, compared to CRYO and RF. However, the availability of a 3D-electroanatomical mapping (EAM) during RF procedures contributed to significantly less X-ray exposure, compared to PFA and CRYO.
2. Pulmonary vein isolation was successfully achieved in all 870 patients; however, first-pass isolation rate was higher with PFA.
3. All ablation strategies showed a low risk of serious complications, ranging between 0.9% and 1.1%. Pulsed field ablation patients trended towards lower overall complication rates (*P* = 0.052); this difference was mainly driven by a significantly higher rate of minor complications among CRYO and RF patients (*P* = 0.032).
4. Long-term arrhythmia freedom was high (>70% at 1 year) and not significantly different among technologies. Pulsed field ablation patients exhibited a trend towards a lower rate of AF as recurrent arrhythmia.
5. At the time of repeat ablation, PFA patients showed a significantly lower number of reconnected PVs compared to those initially treated with CRYO or RF (*P* = 0.01). Right inferior PV reconnection was significantly less frequent among PFA patients (*P* = 0.04), whereas CRYO patients showed a tendency for fewer left superior PV reconnection (*P* = 0.09).

The PVs have been described as the main site harbouring triggers initiating AF paroxysms. Therefore, PVI is the primary treatment strategy in patients undergoing either surgical or percutaneous AF ablation, particularly among PAF cases where the likelihood of the PVs being the culprit sources of ectopic beats initiating AF is significantly higher that non-PAF patients. Yet, durable PVI is difficult to achieve; even after the remarkable technological advancements witnessed in the field of interventional arrhythmia management over the past few years, PV reconnection rates with classic energy sources for cardiac ablation (e.g. radiofrequency and cryothermy) have been reported to be up to 80% in the repeat ablation substudy of the FIRE and ICE randomized trial.^1,4,24–27^

Pulsed field ablation has emerged as a novel energy source to selectively target and ablate cardiac tissue. Tissue selectivity, which significantly reduces the risk of thermal damage to adjacent healthy tissues, in combination with preservation of the extracellular matrix integrity,^28^ makes PFA a versatile and safe option for PVI. One of the key advantages of PFA is that it operates on a non-thermal principle, which sets it apart from traditional thermal ablation methods, such as cryoballoon and focal RF ablation. The only published randomized study to compare PFA with thermal energy sources recently confirmed the non-inferiority of this novel energy source compare to CRYO and RF, with respect to serious adverse event rates and clinical success at 1 year.^10^

An important feature of PFA is that the waveform can be optimized to improve tissue selectivity, as well as lesion depth and durability, by customizing the treatment to the specific characteristics of the target tissue.

In the present study, we performed a comparison among PFA performed via the pentaspline catheter Farawave™, the first device to receive regulatory approval and arguably the most studied PFA technology, and two classic thermal energy sources, namely cryothermy and focal RF for PVI. From a procedural standpoint, PFA contributed to a significantly higher rate of first-pass PVI and shorter procedural and LA dwelling times compared to the two other ablation strategies.

A key factor for the shorter procedural times with PFA is due to the design of the catheter, which allows for easy PV cannulation and catheter manoeuverability in the LA. In a recent multicentre study from our group,^29^ the pentaspline PFA catheter confirmed its versatility, as assessed by objective (procedural times) and subjective (*ad hoc* questionnaire) data supporting a comfortable transition from pre-existing to newer single-shot technologies among experienced operators,^29^ as well as a steep learning curve among operators with limited experience.^13^

Radiation exposure during catheter ablation procedures may have a long-term adverse impact on the patients and, above all, the medical staff, who is chronically exposed to ionizing radiations. An important finding of our study is the significantly shorter fluoroscopy time among RF patients. These results do not seem to be related to the ablation strategy itself, but are likely due to the availability of a 3D-EAM guidance during RF procedures. In this perspective, as the integration between the Farapulse™ system and an EAM system is being developed, the drawback of fluoroscopy dependency of PFA will soon be overcome.

Another finding of our analysis is a comparable safety profile of all technologies for PVI, as confirmed by a rate of major periprocedural complications of ∼1%, mainly represented by major vascular complications. Other serious complications included two (0.23%) cases of cardiac tamponade and two (0.23%) of transient ischaemic attacks in an overall population of 870 PAF patients. Similar findings were observed in the ADVENT randomized trial,^10^ which also documented a 9.1% rate of silent cerebral lesions at post-procedural brain magnetic resonance imaging. These findings are in line or even lower than the available literature on PFA, as well as thermal energy-based ablation.^13,30,31^

Of note, the non-thermal nature of PFA contributed to the reported significantly lower rate of minor complications compared to CRYO and RF groups. This difference was mainly driven by the occurrence of transient PN injury among CRYO patients and chest pain/pericarditis among RF ones, both complications known to be due to thermal effects to surrounding tissues.

From a clinical outcome standpoint, freedom from any atrial tachyarrhythmia ranged between 72% with RF and 79% with PFA at one year. Although there were no significant differences in overall arrhythmia-free survival, a trend towards lower sustained AF episodes was documented with PFA (*P* = 0.09) during follow-up.

In light of the known high rate of PV reconnection after thermal energy-based PVI and the enthusiasm created by a previous phase 3 trial on PFA showing 96% durable PV isolation at invasive remapping,^4,14^ it is likely that other factors are responsible for the observed absence of significant differences in arrhythmia control among groups.

Above all, we believe that, whether PFA provides any additional benefits for arrhythmia control, this will require larger studies and an arrhythmia monitoring strategy based on implantable loop recorders to better appreciate any differences in AF control and residual burden.

Nonetheless, we need to keep in mind that PFA provides a significant advantage over CRYO and RF, as it eliminates any risks of unintended collateral thermal injury to adjacent structures potentially resulting in disabling or life-threatening procedural complications.

Additionally, studies performing a pharmacological induction protocol to systematically map and ablate triggers initiating AF showed that other sources of ectopic beats are elicitable with a rate that is higher in non-PAF patients but still up to 20–30% in PAF, depending on the trigger protocol and the criteria adopted to define them.^22,32^ Left atrial substrate abnormalities (e.g. low voltage areas) have also been described in ∼10%.^33^ These evidences may explain a certain percentage of recurrences resulting from atrial ectopies originating from extrapulmonary site and abnormal atrial substrate, which would contribute to arrhythmia recurrence and maintenance irrespective of durable PVI.

In regard to durable PVI, our analysis demonstrates for the first time that the rate of PV reconnection at repeat procedures is significantly lower among patients receiving PFA at index procedure. Also, significantly fewer reconnected right inferior PVs were documented at post-PFA repeat ablation. In contrast, the left superior was the most frequently reconnected PV after PFA, with a pattern of reconnection consistently involving the anterior aspect and the carina of the vein. Similar findings were previously reported in recent studies focusing on high density mapping in patients with recurrent arrhythmias and a previous PFA procedure with the multispline PFA catheter.^34,35^

## Limitations

This study has several limitations that need to be acknowledged. First, this study is a retrospective review of individual prospective registries, which introduced all the inherent limitations and biases associated with its design. Secondly, the frequency and modality of arrhythmia monitoring might have caused an underestimation of arrhythmia recurrences. Thirdly, our study included only a PFA device and an RF-based mapping and ablation system. Therefore, it would be inappropriate to generalize these observations to other devices. Also, this analysis did not include the latest generation of focal RF catheters capable of very high power, short duration ablation (e.g. QDOT MICRO™ Catheter, Biosense Webster, Diamond Bar, CA, USA).

## Conclusions

In our cohort, PFA contributed to significantly shorter procedural times and fewer minor periprocedural complications. Fluoroscopy time was significantly shorter with RF, as a result of 3D-EAM guidance availability. Follow-up data showed a similar freedom from any atrial tachyarrhythmia among groups, although a higher rate of PV reconnection was documented in post-CRYO and -RF redo procedures. Future randomized trials comparing the safety and efficacy of different ablation strategies are warranted.

## Data Availability

The data underlying this article will be shared on reasonable request to the corresponding author.
